# HnRNP-L promotes prostate cancer progression by enhancing cell cycling and inhibiting apoptosis

**DOI:** 10.18632/oncotarget.14258

**Published:** 2016-12-27

**Authors:** Xumin Zhou, Qi Li, Jincan He, Liren Zhong, Fangpeng Shu, Rongwei Xing, Daojun Lv, Bin Lei, Bo Wan, Yu Yang, Huayan Wu, Xiangming Mao, Yaguang Zou

**Affiliations:** ^1^ Department of Urology, Nanfang Hospital, Southern Medical University, Guangzhou 510515, P. R.China; ^2^ Department of Urology, Peking University Shenzhen Hospital, Shenzhen PKU-HKUST Medical Center, Shenzhen, 518036, P. R.China; ^3^ Department of Stomatology, Nanfang Hospital, Southern Medical University, Guangzhou 510515, P. R.China; ^4^ Department of Urology, Maternal and Health Care Hospital, Longgang District, Shenzhen 518036, P. R.China; ^5^ Department of Urology, Fourth Affiliated Hospital, Guangxi Medical University, Liuzhou 545005, P. R.China; ^6^ Department of Urology, Affiliated Weihai Second Municipal Hospital of Qingdao University, Weihai 264200, P. R.China

**Keywords:** prostate cancer, HnRNP-L, proliferation, apoptosis, molecular mechanism

## Abstract

Expression of the RNA-binding protein HnRNP-L was previously shown to associate with tumorigenesis in liver and lung cancer. In this study, we examined the role of HnRNP-L in prostate cancer (Pca). We found that HnRNP-L is overexpressed in prostate tissue samples from 160 PC patients compared with tissue samples from 32 donors with cancers other than Pca. Moreover, HnRNP-L positively correlated with aggressive tumor characteristics. HnRNP-L knockdown inhibited cell proliferation and promoted cell apoptosis of Pca cell lines *in vitro*, and suppressed tumor growth when the cells were subcutaneously implanted in an athymic mouse model. Conversely, overexpression of HnRNP-L promoted cell proliferation and tumor growth while prohibiting cell apoptosis. HnRNP-L promoted cell proliferation and tumor growth in Pca in part by interacting with endogenous p53 mRNA, which was closely associated with cyclin p21. In addition, HnRNP-L affected cell apoptosis by directly binding the classical apoptosis protein BCL-2. These observations suggest HnRNP-L is an important regulatory factor that exerts pro-proliferation and anti-apoptosis effects in Pca through actions affecting the cell cycle and intrinsic apoptotic signaling. Thus HnRNP-L could potentially serve as a valuable molecular biomarker or therapeutic target in the treatment of Pca.

## INTRODUCTION

Prostate cancer (Pca) is the second most frequently diagnosed cancer and the fifth leading cause of cancer death in males [1]. In recent years, the incidence of Pca has been increasing while the overall fiver-year survival rate has been decreasing [2]. There is thus an urgent need to understand the processes promoting tumorigenesis and progression in Pca as a means of improving treatment.

Heterogeneous nuclear protein L (HnRNP-L) is one of the hnRNPs family members [3]. It is an abundant nuclear protein that has been identified as a global splicing regulator [4]. It participates in splicing, transportation and degradation of precursor mRNAs [5–9]. Several reports have brought to light the possibility that some splicing factors act as oncogenic or anti-oncogenic factors [10–13]. What's more, HnRNP-L can reportedly regulate the tumorigenic capacity of lung cancer [14, 15]. Furthermore, using two-dimensional electrophoresis and mass spectrometry, Zhao et al. showed that HNRNP-L is differentially expressed in Pca [16]. However, the specific roles of HnRNP-L in Pca remain unclear. To address that issue, we investigated the effects of HnRNP-L expression on tumorigenesis and progression in Pca.

## RESULTS

### HnRNP-L is upregulated in Pca

**Figure 1:**
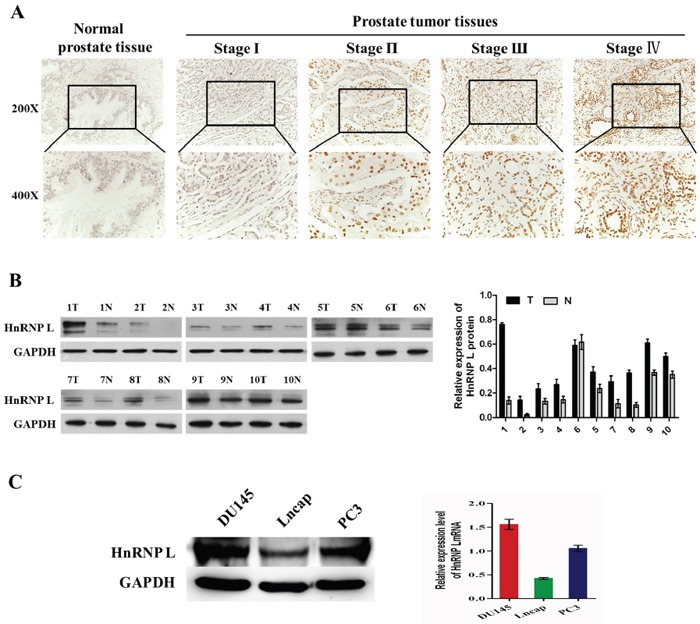
HnRNP L is upregulated in prostate cancer **A**. Expression of HnRNP-L in a tissue microarray containing several prostate cancer and non-prostate cancer tissues (n=192). Representative images of HnRNP-L in Normal and Pca specimens examined by IHC. HnRNP-L was positively detected in Pca tissues (stage I-IV), while it was only weakly or negatively detected in non-prostate cancer tissues. **B**. Expression of HnRNP-L protein in each of the primary PCA (T) and adjacent noncancerous tissues (N) paired from the same patient by western blotting (left) (n=10). The protein expression levels were quantified by comparing the gray level of each band using Quantity One Software. **C**. Western blotting (left) and Real-time PCR (right) show the expression levels of HnRNP-L in DU145, Lncap, PC3 cell lines.

We performed IHC analysis to determine the expression of HnRNP-L in 160 prostate cancer tissues and 32 non-prostate cancer tissues. HnRNP-L was highly expressed in Pca tissues. In contrast, the expression of HnRNP-L was obviously decreased or not detected in non-prostate cancer tissues (Figure 1A). The percentage of nucleoplasm expressing HnRNP-L gradually increased from 34.4% in NC to 78.1% in Pca (*P*<0.001). Among 160 Pca tissues, the expression of HnRNP-L was negatively associated with the clinic pathological parameter: The positive rate of HnRNP-L was significantly increased in low pathological tissues, compared to high pathological tissues (*P*<0.001). At the same time, HnRNP-L positivity was more frequent in Pca patients with more advanced stages (*P*=0.005) (Table 1).

**Table 1:** Correlations between HnRNP-L expression and clinic pathological parameters of Pca samples

Variable	No. of patients	HnRNP L expression		*P*-Value
		Positive	%	
Characteristics				<0.001*
Normal	32	11	34.4	
PCA	160	125	78.1	
Grade				<0.001*
**I**	0	0	0	
**II-III**	78	51	65.4	
**III**	82	74	90.2	
Stage				=0.005*
**I**	14	8	57.1	
**II**	74	52	70.3	
**III-IV**	72	65	90.3	

* *P*-Value of *P*<0.05 were considered statistically siginificant.

In addition, Western blotting was performed to examine the expression of HnRNP L in 10 cases of Pca tissues paired adjacent non-cancerous tissues and 3 Pca cells (DU145, PC3 and Lncap). Western blotting results shown that HnRNP L expression was significantly higher than those in paired adjacent non-cancerous tissues (Figure 1B). At the same time, our results revealed that endogenous HnRNP L was highly expressed in DU145 and PC3 cell lines compared with Lncap cell line (Figure 1C).

### HnRNP-L overexpression promotes the proliferation of prostate cancer cell lines via accelerating the progress of cell cycle

**Figure 2:**
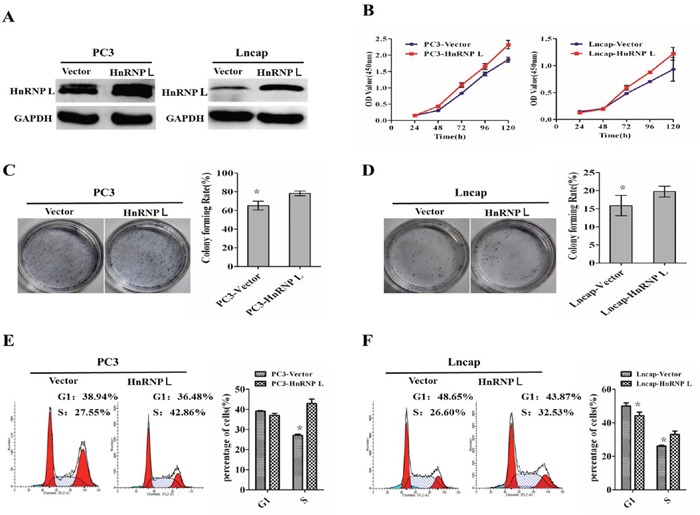
Upregulation of HnRNP-L promotes the proliferation of prostate cancer cell lines via accelerating the progress of cell cycle **A**. Ectopic expression of HnRNP-L in PC3 cells (left) and Lncap cells (right) analyzed by western blotting. GAPDH was used as a loading control. **B, C** and **D**. Overexpression of HnRNP-L promotes the proliferation of PC3 and Lncap cells as detected by CCK-8 assays (B) and colony formation assays (C and D). Each bar represents the mean ± SD of three independent experiments. *:*P*<0.05. **E** and **F**. Flow cytometric analysis shows the accelerating progress of cell cycle after overexpression of HnRNP-L in PC3 and Lncap cell lines. Data represented as mean ± SD of three independent experiments. *:*P*<0.05 (vs. control).

To gain insight to the possible role of HnRNP-L in human Pca cells growth, stable HnRNP-L expressed cell lines were made (Figure 2A). CCK-8 assay showed that HnRNP-L overexpression increased the proliferation of PC3 and Lncap compared with the negative control, respectively (Figure 2B). At the same time, colony formation assay indicated that PC3/HnRNP-L and Lncap/HnRNP L formed more colony numbers than the control groups (Figure 2C and 2D). Regarding the impact on the cell cycle, we performed flow cytometry. After analyzing the cell cycle distribution, we found that HnRNP-L overexpression impeded progression into the S phase in PC3 and Lncap cells when compared with negative controls (Figure 2E and 2F).

### HnRNP L knockdown inhibits the proliferation of prostate cancer cell lines via cell cycle arrest

**Figure 3:**
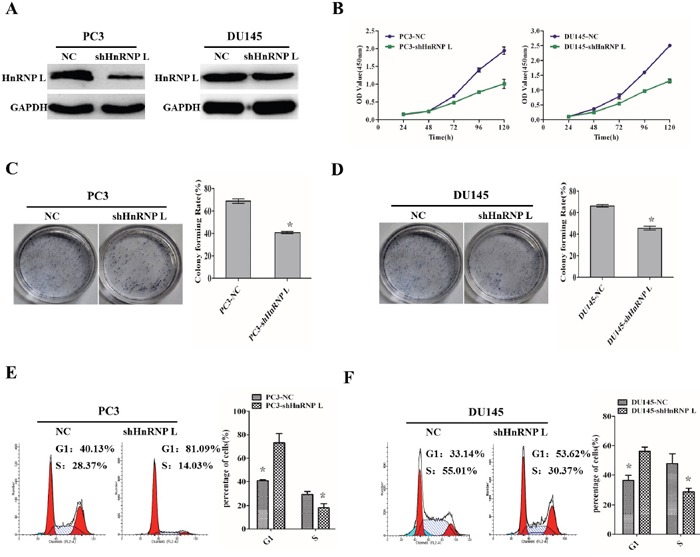
HnRNP L knockdown inhibits the proliferation of prostate cancer cell lines via cell cycle arrest **A**. Ectopic expression of HnRNP-L in PC3 cells (left) and DU145 cells (right) analyzed by western blotting. GAPDH was used as a loading control. **B, C** and **D**. HnRNP-L knockdown inhibits the proliferation of PC3 and Lncap cells as detected by CCK-8 assays (B) and colony formation assays (C and D). Each bar represents the mean ± SD of three independent experiments.*:*P*<0.05. **E** and **F**. Flow cytometric analysis of cell cycle arrest after treatment of shRNAs and negative controls in PC3 and Lncap cell lines. Data represented as mean ± SD of three independent experiments. *:*P*<0.05 (vs. control).

To further reveal the impact of HnRNP-L on Pca proliferation, we stably knocked down HnRNP-L in PC3 and DU145 cells (Figure 3A). Compared with the control groups, knockdown of HnRNP L (shHnRNP-L) expression decreased the proliferation of PC3 and DU145, which was certified by CCK-8 assay (Figure 3B). We also performed the colony formation assay to ensure the effect. We found that PC3/shHnRNP-L and DU145/shHnRNP-L formed fewer colonies than that of control groups (Figure 3C and 3D). Meanwhile, the flow cytometry analysis showed that shHnRNP-L caused cell cycle arrest in G1 phase in PC3 and DU145 cells (Figure 3E and 3F), which partly account for the suppressed proliferation.

### Ectopic expression of HnRNP-L affects tumorigenesis in PC3 cells in nude mice

**Figure 4:**
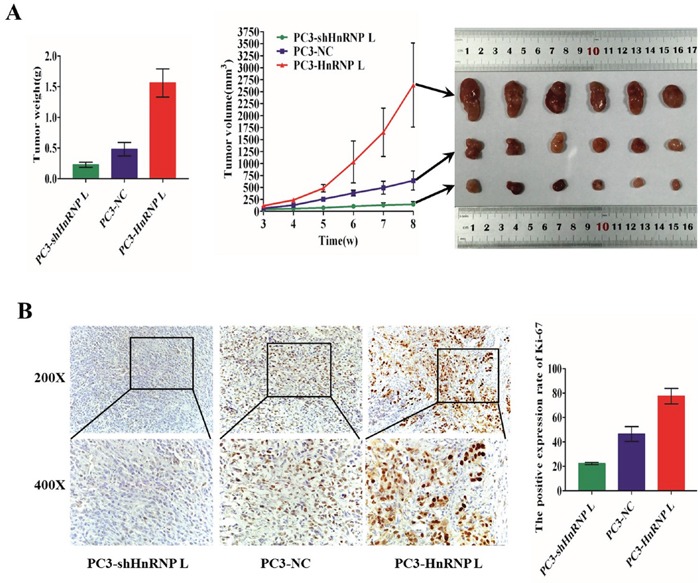
Ectopic expression of HnRNP-L affects tumorigenesis of PC3 cells *in vivo* **A**. Image of tumors dissected from 18 nude mice (n=6 for each group, right). Tumor volumes measured on the indicated days. Data points are the mean tumor volume ± SD (middle). The tumors weight were weighed and analyzed by F-test (left). Means ± SD were shown(n=6).*:*P*<0.05 (vs. control). Overexpression of HnRNP-L promotes the tumor growth, whereas HnRNP-L knockdown inhibits it in nude mice. **B**. Representative immunohistochemistry staining images of Ki-67 from sample tumor tissues in each group (left). Mean positive rates ± SD were shown(n=6). *:*P*<0.05 (vs. control) (right).

To investigate the effect of HnRNP-L on tumor growth, we performed tumorigenesis assays in nude mice. PC3/HnRNP-L, PC3/shHnRNP-L and the negative control cells were injected subcutaneously into the right armpit regions of each mouse, respectively. After injection 4 weeks, xenograft tumors were grew up in all mice. We could found that the group of PC3/HnRNP-L formed tumors much faster than that of negative control group, while the PC3/shHnRNP-L group grew tumors more slowly than the negative group (Figure 4A). Furthermore, we used immunohistochemical analysis to identify the expression of ki-67 in paraffin-embedded mice tumors. As shown in Figure 4B, the expression of ki-67 in HnRNP-L overexpressed group was significantly higher than that in control group. Ki-67 expression was lower in the HnRNP-L knockdown group than the control group. All these results demonstrated that HnRNP-L overexpression promoted the tumor growth while HnRNP-L knockdown inhibited it in nude mice.

### HnRNP L affects the cell cycle through binding to p53 mRNA in PC3 cells

**Figure 5:**
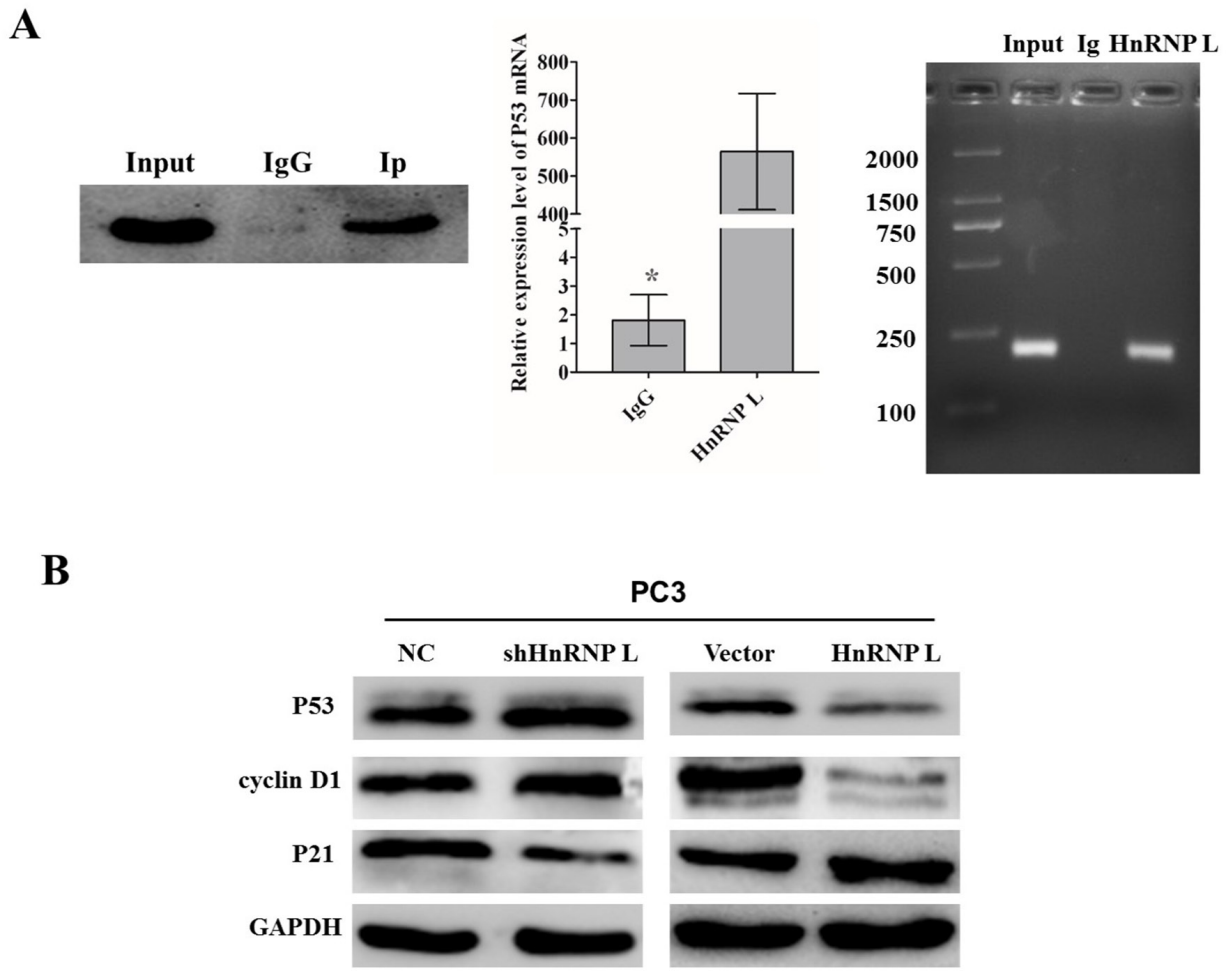
Identification of HnRNP-L as a p53 mRNA-binding protein **A**. Adequate cytoplasmic extracts were incubated with a monoclonal antibody for HnRNP-L or IgG overnight before the purification of RNA and then western blotting was performed to identify the efficiency of co-immunoprecipitation. IgG was served as negative control (left). P53 mRNA identified as the co-purified mRNA with HnRNP-L was proven by immunoprecipitation followed by RT-PCR. PC3 cytoplasmic fractions were immunoprecipitated with anti-HnRNP-L or IgG and the co-immunoprecipitated mRNA were extracted for Real time PCR (middle). The PCR products were separated by 2.0% agarose gel electrophoresis. The amplification of the cDNA from the input was shown as positive control (right). **B**. Western blotting analysis of the expression of p53 and other cell cycle-related proteins, such as cyclin D1 and p21. GAPDH was used as a loading control.

By means of bioinformatics prediction, we found that HnRNP-L could integrate with some mRNA sites of the tumor suppressor p53, which was a primary regulator of cell cycle activity. To further confirm the interaction between HnRNP-L and p53 mRNA, RNA-binding protein immunoprecipitation (RIP) with HnRNP-L antibody and IgG was performed to pull down the P53 mRNAs, respectively; and then these mRNAs were analyzed by RT-PCR. QRT-PCR results showed that the ratio of co-purified p53 mRNA in the HnRNP-L antibody group to the IgG group was 312. Then, 2.0% agarose gel electrophoresis was used to separate the PCR products. As shown in Figure 5A, p53 cDNA was obviously detected in the PCR product immunoprecipitated by hnRNP-L, while was hard to detect in those immunoprecipitated by IgG. These results demonstrated that HnRNP-L surely interacted with p53 mRNA.

To deeply elucidate the underlying molecular mechanisms accounting for the effect in cell cycle triggered by HnRNP-L, we examined the expression of P53 and other cell cycle-related proteins, such as cyclin D1 and p21. The results indicated that the overexpression of HnRNP-L led to an increased expression of P53 and cyclin D1 and decreased expression of p21, on the contrary, HnRNP-L knockdown led to the opposite effect in PC3 cells (Figure 5B). Therefore, these results demonstrated that HnRNP-L might be capable of regulating the progression of cell cycle, which contributed to the promoting effect on cell proliferation in Pca cells.

### HnRNP-L regulates the apoptosis of PC3 cells via interacting with BCL-2

**Figure 6:**
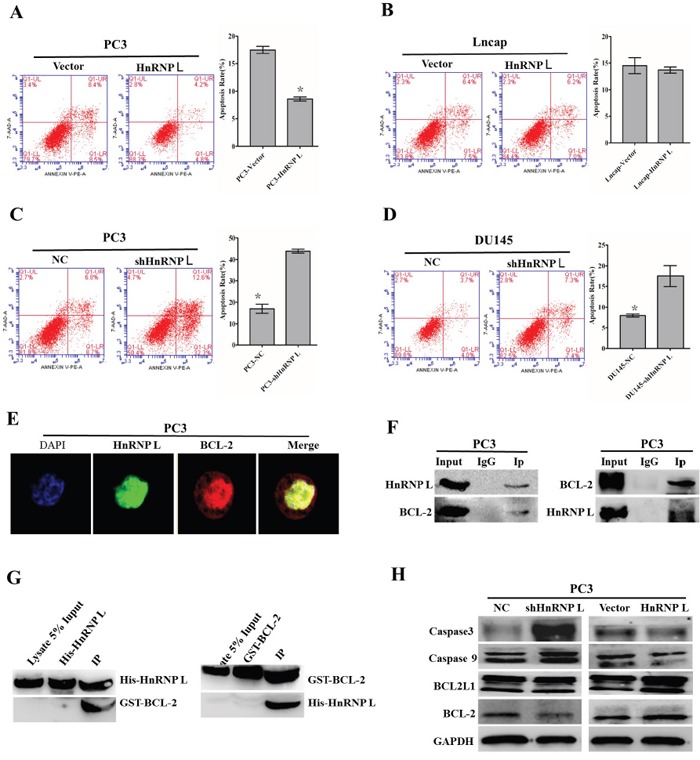
Effect of HnRNP-L aberrant expression on prostater cancer apoptosis **A, B, C** and **D**. Overexpression of HnRNP-L inhibits the apoptosis of PC3 (A) and Lncap (B) cells, whereas HnRNP-L knockdown promotes the apoptosis of PC3 (C) and DU145 (D) cells, which were analyzed by flow cytometry using Annexin V-FITC/PI staining. Statistical analysis of flow cytometry results. The presented columns are given as the means ± SD. *:*P*<0.05 (vs. control). **E**. Co-localization of HnRNP-L with BCL-2 in PC3 cells. PC3 cells were immunostained for HnRNP-L (green) and BCL-2 (red), followed by con-focal-microscopic analysis of their co-localization. **F**. Reciprocal co-immunoprecipitation assays of HnRNP-L and BCL-2 in PC3 cells, which were performed *in vitro*. IgG group was served as IP antibody control. **G**. Pull down assays of His-tagged HnRNP-L and GST-tagged BCL-2, which were induced to express abundantly in Rossatte bacteria. The input group and tag-purification (His tag or GST tag) group were treated as negative control in this experiment. **H**. Western blotting was used to exam the expression of BCL-2, caspase 3 and caspase 9 after the ectopic expression of HnRNP-L. GAPDH was served as a loading control.

We then examined the involvement of HnRNP-L in the apoptosis of prostate cancer cells through knockdown and overexpression of HnRNP-L. As shown by flow cytometry analysis, we found that PC3/shHnRNP-L and DU145/shHnRNP-L significantly increased the apoptotic rates as compared with that of negative groups (Figure 6A and 6B); in contrast, HnRNP-L overexpression in PC3 led to the decreased rates of both early and late apoptotic cells compared to mock transfection; however, this effect was not obvious in Lncap cells (Figure 6C and 6D).

To explore the interactions between HnRNP-L and bcl-2, firstly, we identified their interaction sites in subcellular fractions of PC3 cells (middle expression of HnRNP L). Localizations of HnRNP-L and bcl-2 were detected using indirect immunofluorescence confocal microscopy. The results showed that HnRNP-L and bcl-2 were co-localized in the nucleoplasm in PC3 cells (Figure 6E). And then, we carried out co-immunoprecipitation assay to confirm the interaction between HnRNP-L and bcl-2 *in vitro*, and we found that bcl-2 was co-purified with the HnRNP-L antibody while the IgG was not. On the other hand, the HnRNP-L specific bands also appeared in the bcl-2 antibody purified group without in the IgG group (Figure 6F and 6G). All in all, these results revealed that HnRNP-L interacted with bcl-2 in PC3 cells.

To our knowledge, bcl-2 was closely related with caspase family in the intrinsic apoptotic pathway. In this study, western blotting was performed to detect the expression of bcl-2, caspase 3 and caspase 9 after ectopic expression of HnRNP-L. As shown in Figure 6H, the expression of bcl-2 was downregulated and the expression of caspase 3 and caspase 9 was upregulated followed by HnRNP-L knockdown, while the opposite effect appeared after overexpression of HnRNP-L in PC3 cells. All these results indicated that the activation of intrinsic apoptotic pathway triggered by HnRNP-L was involved in Pca cells apoptosis.

## DISCUSSION

HnRNP-L was originally identified as a RNA-binding protein that participated in the formation, packaging, processing, and function of mRNA [4]. HnRNP-L is only weakly expressed in normal tissues, but is abnormally overexpressed in diverse human cancers, including lung, liver, ovarian, colorectal and breast cancers [14, 17–20]. In addition, studies revealed that HnRNP-L regulated cancerogenic capacity by interacting with downstream mRNA [14, 17]. On the other hand, little was known about the actions of HnRNP-L in Pca. In the present study, we demonstrated that HnRNP-L expression was upregulated in Pca tumor samples, as compared to matched adjacent normal tissues. What's more, high expression of HnRNP-L was positively associated with aggressive characteristics. These findings suggest that HnRNP-L expression may be treated as a novel marker to identify patients with Pca cancer.

The effects of altering HnRNP-L expression provide further functional evidence of HnRNP-L-mediated Pca proliferation and inhibition of apoptosis *in vitro* and *in vivo*. Tumor progression of reflects dysregulation of cell apoptosis and/or the cell cycle, which results in the unlimited proliferation in cancer [21, 22]. The major regulatory checkpoint in this process is the transition of cell cycle from G1 phase to S phase. Cell cycle transition is characterized by interaction of cyclins and CDK inhibitors [23]. In our research, flow cytometric analysis revealed that altering HnRNP-L expression affected the cell cycle, most likely through HnRNP-L binding to p53 mRNA, which we verified in Pca cell lines. We also evaluated the expression of proteins involved in regulating cell cycle transition from G1 phase to S phase. Our results showed that overexpression of HnRNP-L inhibited expression of p53 and p21 while enhancing expression of cyclin D1, and that HnRNP-L knockdown had the opposite effect. The tumor suppressor p53 is a primary regulator of cell cycle activity that induces cell cycle arrest and apoptosis through induction of p21WAF1 [24]. As a cyclin-dependent kinase inhibitor (CdkI), p21WAF1, along with p27 and p57 interact the Cdk-cyclin complex. The group is regulated both by internal and external signals, with p21WAF1 expression under transcriptional control of the p53 tumor suppressor gene [25]. Cyclin D1 also plays crucial roles in this process by activating other cell cycle factors [26, 27]. These data shed light on the way in which HnRNP-L promotes proliferation of Pca cells. HnRNP-L could potentially serve as a therapeutic marker for Pca.

HnRNP-L is reportedly linked to the caspase family, which are absolutely necessary for the initiation and execution of cell apoptosis [14, 15, 28–30]. We also learned that apoptosis in Pca cells is associated with the activation of caspase 3, which is an important effector in the intrinsic signaling pathway [31–33]. In addition, Kang et al. confirmed that HnRNP LL, a paralog of HnRNP-L, bound to the BCL-2 i-motif, and that the transcriptional complex between the BCL-2 i-motif and HnRNP LL acts as a molecular switch for control of gene expression that can be modulated by small molecules [34]. Interestingly, BCL-2 is an important regulator downstream of caspase signaling [35]. In this study, we showed that HnRNP-L interacts with BCL-2. Moreover, the observation that HnRNP-L knockdown increased apoptosis among Pca cells, while HnRNP-L overexpression reduced the rate of Pca cell apoptosis via the intrinsic signaling pathway, as indicated by altered expression of BCL-2, caspase 9 and caspase 3. This suggests HnRNP-L is involved in modulating the bcl-2/caspase9/caspase3 signaling pathway.

In summary, results from our study demonstrate that HnRNP-L is overexpressed in Pca, suggesting it may be a useful therapeutic target for Pca. HnRNP-L appears to exert pro-proliferation and anti-apoptosis effect in Pca. These actions of HnRNP-L in Pca are most likely mediated via the p53/p21/cyclin D1 and BCL-2/caspase 9/caspase 3 signaling pathways. However, further investigation will needed to identify other functions of HnRNP-L and the potential mechanisms by which it acts in Pca.

## MATERIALS AND METHODS

### Clinical specimens

In our study, we examined the expression of HnRNP-L using a tissue microarray (Alenabio, xi’an, China) containing several prostate cancer and normal prostate tissues, which was constructed from 192 patients. The experiment was performed with the immunohistochemistry assay. Furthermore, the correlation of HnRNP-L expression and clinicopathological parameters was further identified using certain statistical analysis.

### Cell cultures

Human prostate cancer (Pca) cell lines PC3, DU145 and Lncap were derived from American Type Culture Collection. Cells were cultured in complete medium (1640 medium [Gibco, China] was supplemented with 10% FBS [Sangon, China]) and incubated under 5% CO_2_ at 37°C.

### Establishment of stably transfected cell lines

The construction of slow virus vector was done in Shanghai Ji Kai Gene Technology Co., Ltd. For HnRNP-L overexpression, full-length human HnRNP-L cDNA was amplified by polymerase chain reaction, and subcloned into the pBaBb-puromycin plasmid. For deletion of HnRNP-L, the short hairpin RNA targeting HnRNP-L (shHnRNP-L) were cloned into the pSUPER-retro-puromycin plasmid. The shHnRNP-L targeting sequence was: sense, 5′-UUCUCCGAACGUGUCACGUTT-3′, antisense, 5′-AUCUUAGAUUGAUCCAAGCTT-3′. Plasmids combined with PIK vector or blank pBaBb-vector were generated as negative controls. After transfection 48 hours, cells were treated with 3 ug/mL puromycin for 2 weeks. Then, cells with stable HnRNP L overexpression and knockdown were selected for subsequent experiment.

### Real-time RT-PCR and western blotting analysis

Total RNA was extracted using TRizol reagent (TAKARA, China), and first-strand cDNA was synthesized with the PrimeScript RTReagent Kit (TAKARA, China) according to the manufacturer's recommended instructions. Reverse Transcription was carried out with the SuperScript First-Strand Synthesis System for RT-PCR (Invitrogen, Carlsbad, CA) according to the manufacturer's protocol. Real-time RT-PCR was carried out using complementary DNA and SYBR-Green II (TAKARA, China). The data were normalized to the housekeeping gene GAPDH and counted using the 2^−ΔΔCT^ method. RT-PCR was performed on the ABI PRISM 7500 Sequence Detection System (Applied Biosystems) and repeated at least three times. Primers were designed using Primer Express software, and their sequences were: HnRNP-L, sense, 5′-TTGTGGCCCTGTCCAGAGAATT-3′; anti-sense, 5′-GTTTGTGTAGTCCCAAGTATCCTG-3′; GAPDH, sense, 5′-AGAAGGCTGGGGCTCATTTG-3′; anti-sense, 5′-AGGGGCCATCCACAGTCTTC-3′.

Cells or tissues were washed three times by cold PBS and lysed by lysis buffer (KaiJi, China) containing protease inhibitor cocktail. The concentration of protein was determined by bicinchoninic acid method. For Western blotting analysis, proteins were separated by 8%~12% SDS-polyacrylamide gel and was then transferred onto 0.45 μm or 0.22 μm pore-size PVDF membrane. After blocking in 5% skimmed milk in TBST, the membrane was incubated with appropriate primary antibodies at 4°C overnight. Then, the membrane was washed 6minutes for five times, followed by incubation with HRP-linked secondary antibody for 1 hour at room temperature. GAPDH was used as an internal control. Protein blots were detected by exposing chemiluminescent HRP substrate to film.

### Cell viability analysis

After trypsinization, 2×10^3^ cells were seeded into 96-well plates and allowed for culture overnight. The vitalities of cell proliferation were determined by Cell Counting Kit-8 (CCK-8) assay (DoJinDo, Cat.No.CK04) according to the manufacturer's protocol on days 1, 2, 3, 4 and 5. In briefly, 10μl CCK-8 solution was added into each plate, and then the absorbance was measured at 490 nm after 2 h of incubation at 37°C using a microplate reader. Three duplicate wells should be plated for each cell group.

### Colony formation assays

To identify the ability of stably transfected cells to form colonies, 1000 cells were seeded into 6-cm culture dishes and incubated for 2 weeks. The complete medium was changed every 3-4 days. After 2 weeks, cells were washed three times with cold PBS, and then fastened with 4% paraformaldehyde for 30 minutes. The colonies were stained with hematoxylin for 30 minutes, and then counted using a microscope.

### Cell-cycle analysis

The stably transfected cells for growth phase analysis were harvested in PBS and fixed in 70% ice-cold ethanol for overnight at 4°C. After washed in PBS, the cells were stained with 200μl propidium iodide (Reagent A) for 30 min the next day. The cell cycle phases were detected by flow cytometry system (BD FACSCalibur).

### Cell apoptosis analysis

To analyze whether HnRNP-L regulates the apoptosis, the stably transfected cells were harvested and stained with annexin V-FITC and propidium iodide (PI) according to the manufacturer's instructions (MULTI SCIENCES, AP101). The apoptotic percentages of the stably transfected cells were analyzed by flow cytometry using a BD FACSCalibur system.

### Tumorigenesis *in vivo*

All of the male athymic mice (4 weeks) were gained from the experimental animal center of southern medical university (GuangZhou, China) and fed under specific pathogen free conditions. What's more, all the experimental operations were performed in accordance with the institutional ethical guidelines. PC3 cell line was used to establish prostate cancer xenograft. Combined with the results of previous experiments and the consideration of animal experimental cost, we divided these mice into three groups: the lentiviral-mediated HnRNP-L stably overexpressed group (n=6), the HnRNP-L stable knockdown group (n=6) and the control group (n=6). A dose of 5×10^6^ PC3 cells suspended in PBS were injected subcutaneously into the right armpit regions of each mouse. After 8 weeks, all mice were sacrificed and tumor weight and tumor volume were measured respectively.

### Immunofluorescence assays

Cells were cultured in confocal dishes for 24 h and fixed with 4% paraformaldehyde for 40 minutes. After washed three times with PBS, cells were permeabilized with 0.3% Triton X-100 for 15 minutes and blocked with 5% BSA for 1 hour at room temperature. Then, cells in confocal dishes were incubated with HnRNP L and BCL-2 primary antibodies overnight at 4°C and incubated with appropriate secondary antibodies (ZSGB-BIO) for 1.5 hours at room temperature. Finally, cells were counter-stained with DAPI for 5minutes and detected under a confocal laser-scanning microscope.

### Immunohistochemistry and evaluation criteria

For immunohistochemistry (IHC), prostate tissues were fixed with 10% formalin, dehydrated and embedded in paraffin. Then 5-μm sections were prepared with a microtome. The sections were deparaffinized, rehydrated and the endogenous antigen was retrieved by autoclave for 8 minutes in citric-acid buffer (10 mM citrate buffer, PH8.0). Then slides were incubated with anti-HnRNP and anti-Ki-67 primary antibody at 4°C overnight and then HRP conjugated secondary antibody at room temperature for 30 minutes. Signal was detected with DAB and nuclei were counterstained with hematoxylin. HnRNP-L expression in cancer cells of the prostate cancer tissues was evaluated by the total scores of intensity and the percentage of stained cells. Intensity of stained cells was scored as 0—lack of staining (−), 1—weak staining (+), 2—moderate staining (++) and 3—strong staining (+++). The percentage of stained cells was recorded as 1—25% or less, 2—26% to 50%, 3—51% to 75% and 4—greater than 75%. Total scores of stained cells ranged from 0 to 12. All sections were defined as having low expression—0 to 7 or high expression—8 or greater by semiquantitative score. Slides were assessed by 2 pathologists.

### Co-immunoprecipitation and pull down

For the immunoprecipitation assay, 8×10^7^ PC3 cells were harvested and lysed by 600μL lysis buffer (KaiJi, China) supplemented with PMSF (1:100). Half amounts of protein (>500μg) were incubated with 5μL anti-HnRNP L (Abcam, ab6106), anti-bcl-2 (abclone) or 2μL IgG antibody (Univ-Bio, ShangHai, China) at 4°C overnight. 50μL protein A/G beads were washed by RIP wash buffer twice and then added to the mixture on the next day and incubated for 16 hours at 4°C. Then we used SDS/PAGE and western blot to identify the co-purified proteins. IgG group was used as negative control to eliminate unspecific associations.

For the pull down assay, two plasmids, His-tagged HnRNP-L and GST-tagged BCL-2, were successfully constructed. Then we transformed these two plasmids into Rossatte bacteria (Biowit Technologies, ShenZhen, China) and used appropriate concentrations of IPTG to induce the over expression of HnRNP-L and bcl-2, respectively. Adquate of bacteria Liquid were gathered and centrifuged. Then the bacteria depositions were lysed by lysis buffer (KaiJi, China) containing protease inhibitor cocktail. Take 10μl lysate and set aside on ice as input control. Then divide the lysate into two portions of equal volume for tag-purification group and pull-down group. Add the tag beads to both of these two groups and incubate for 8 hours at 4°C with gentle rotation. Then add the equal volume of another lysate into the pull-down group and incubate overnight at 4°C with gentle rotation. The next day, the beads of these two groups were washed by ice-cold PBS four times. Then we used SDS/PAGE and western blot to identify the protein which was pulled down by another. The input group and tag-purification group were treated as negative control in this experiment.

### RNA-binding protein immunoprecipitation (RIP)

The procedures were carried out in an RNase-free environment. 1×107 cells were harvested and resuspended in PBS and nuclear isolation buffer. Nuclear pellets lysis was centrifuged and resuspended in RIP buffer. Then, nuclear pellets lysis was divided into two fractions of 500μl each (for Mock and IP) and sheared on ice using a dounce homogenizer. Pellet nuclear membrane and debris were obtained by centrifugation at 14,000 rpm for 10 min at 4°C. Primary antibody and IgG were added into the beads, respectively, and incubated overnight at 4°C with gentle rotation. After washed with PBS, 100μl freshly ice-cold RIP buffer was resuspended with the beads and incubated for 2 hours at 4°C with gentle rotation. Protein isolated by the beads would be detected by western blot analysis, and these unbound materials would be taken off. RNA that was bound to immunoprecipitated RBP would be purified by TRIzol RNA extraction reagent (1 ml) according to manufacturer's instructions. Finally, RNA was reverse transcribed into cDNA and detected by DNA electrophoresis analysis.

### Statistical analysis

All of the experiments were repeated at least three times and the data were showed as means ± S.D. The student's t-test or one-way ANOVA were used to compare the differences between two independent samples. The correlation between HnRNP-L and the other clinical characteristics was assessed using Pearson linear-regression analysis. All statistical analyses were carried out with SPSS 13.0. *P*<0.05 was considered statistically significant.
